# Three-year experience with the Sophono in children with congenital conductive unilateral hearing loss: tolerability, audiometry, and sound localization compared to a bone-anchored hearing aid

**DOI:** 10.1007/s00405-016-3908-6

**Published:** 2016-02-29

**Authors:** Rik C. Nelissen, Martijn J. H. Agterberg, Myrthe K. S. Hol, Ad F. M. Snik

**Affiliations:** 1 Department of Otorhinolaryngology, Donders Institute for Brain, Cognition and Behaviour, Radboud University Medical Center, P.O. Box 9101, 6500 HB Nijmegen, Netherlands; 2 Department of Biophysics, Donders Institute for Brain, Cognition and Behaviour, Radboud University, Nijmegen, Netherlands

**Keywords:** Transcutaneous implant, Percutaneous implant, BAHA, Bone-anchored hearing implant, Hearing loss, Aural atresia

## Abstract

Bone conduction devices (BCDs) are advocated as an amplification option for patients with congenital conductive unilateral hearing loss (UHL), while other treatment options could also be considered. The current study compared a transcutaneous BCD (Sophono) with a percutaneous BCD (bone-anchored hearing aid, BAHA) in 12 children with congenital conductive UHL. Tolerability, audiometry, and sound localization abilities with both types of BCD were studied retrospectively. The mean follow-up was 3.6 years for the Sophono users (*n* = 6) and 4.7 years for the BAHA users (*n* = 6). In each group, two patients had stopped using their BCD. Tolerability was favorable for the Sophono. Aided thresholds with the Sophono were unsatisfactory, as they did not reach under a mean pure tone average of 30 dB HL. Sound localization generally improved with both the Sophono and the BAHA, although localization abilities did not reach the level of normal hearing children. These findings, together with previously reported outcomes, are important to take into account when counseling patients and their caretakers. The selection of a suitable amplification option should always be made deliberately and on individual basis for each patient in this diverse group of children with congenital conductive UHL.

## Introduction

Several studies have demonstrated delays in development and school performance in children with unilateral hearing loss (UHL) compared to children with normal hearing [1–3]. A specific condition of UHL is congenital conductive UHL, which is often caused by anatomical anomalies, such as aural atresia and/or ossicular chain anomalies. These relatively rare abnormalities affect approximately one in 10,000 live births [4]. Depending on the severity of the anomaly, several treatment options are to be considered in children presenting with such altered anatomy: surgical correction of the anomaly if feasible, amplification by means of a conventional hearing aid if possible or a bone conduction device (BCD) in all other cases, or conservatively monitoring the child’s development. When intervention is required, amplification of sound by means of a percutaneous BCD is associated with better audiological outcome than surgical intervention, especially in patients with considerable anatomic deviations [5–7].

In 2011, a new passive transcutaneous BCD (Sophono Alpha 1) has been introduced [8]. The Sophono provides hearing amplification through an intact skin due to magnetic coupling, in contrast to the well-established percutaneous BCDs, most commonly known as bone-anchored hearing aids (BAHA), currently marketed as Cochlear’s Baha system and Oticon Medical’s Ponto system. In a previous study [9], six children with congenital conductive UHL who used the Sophono were compared with six children with congenital conductive UHL who used a BAHA. The BAHA was more powerful; measurements with a skull simulator indicated a 10 dB higher output than with the Sophono. Furthermore, patients who used a BAHA demonstrated better aided thresholds than those using a Sophono. Another study reported that aided thresholds in patients using the Sophono were comparable to those of the same patients using a BCD on a softband [10]. Implant loss and soft tissue problems are expected to be encountered less frequently with the Sophono. This is especially relevant for the application of BCDs in children, as it is reported that children using a BAHA with a previous generation implant experience more complications and implant losses compared to adults [11]. However, it should be noted that, owing to new and wider diameter implant designs, soft tissue reactions of BAHAs have decreased significantly in adults [12, 13], while implant survival increased in the pediatric population [14].

In the current study, we report on soft tissue tolerability, hearing results, and sound localization abilities of the same six patients with congenital conductive UHL implanted with the Sophono, that have been described in a previous study [9]. The outcomes are compared to a group of six children with congenital conductive UHL that use a BAHA. The audiometric outcome of an updated version of the sound processor (Alpha 2) was investigated. Sound localization was tested elaborately.

## Methods

### Ethical considerations

Assessment by the local ethics committee was not required as the application of BCDs is a regular health care treatment option in the Netherlands. Furthermore, all testing data were used in retrospect and part of regular follow-up measurements, which were used to optimize outcomes with the respective BCDs.

### Patients and BCDs

Retrospectively, the follow-up data of the same six patients implanted with the Sophono who participated in a previous study [9] were gathered. Five patients (patients 1–5) suffered from high-grade unilateral congenital aural atresia type IIb or III (according to Cremers’ classification [15]) and one patient suffered from a congenital ossicular chain anomaly (patient 6), resulting in severe conductive hearing loss in the impaired ear. The contralateral, anatomically unaffected ears had near normal hearing thresholds. In all cases, surgery was not considered to be a suitable treatment option. The caretakers of the patients were given the possibility to choose the Sophono instead of the BAHA after elaborate counseling. Outcomes were compared to six patients with aural atresia type III that used a BAHA who also participated in the same previous study [9].

The Sophono Alpha 1 (Sophono, Inc., Boulder, CO, USA; recently acquired by Medtronic, Inc., Fridley, MN, USA) is a BCD that is magnetically coupled to surgically implanted double magnets through an intact skin. Implant surgeries were performed between April 2010 and December 2011. The applied surgical technique has been described in detail [8]. Recently, the sound processor was updated (Alpha 2) and aided thresholds with this new sound processor were tested in the current manuscript. The even more recent Alpha 2 MPO was not yet made available to the study population at the time of the measurements.

All patients in the BAHA group were implanted with the Baha 3.75-mm-diameter flange fixture (Cochlear Bone Anchored Solutions AB, Mölnlycke, Sweden). Implant surgery was performed between October 2008 and October 2011 according to the Nijmegen linear incision technique [16] and in two phases for all patients under 10 years old. The Divino sound processor was used by five patients (patients 7, 8, 10, 11, and 12) and the BP100 by one patient (patient 9).

### Tolerability and appreciation

During regular follow-up visits of at least one visit per year, the local soft tissue status was monitored. Skin reactions were recorded according to Holgers’ classification [17]. Furthermore, information on the actual usage of the device was surveyed, i.e., frequency and duration of the wearing time, and general satisfaction was enquired.

### Audiometry

The patients with the Sophono underwent audiometric evaluation for the aided condition with the first-generation (November 2012) and the updated sound processor (May 2013). For the BAHA patients there were no more recent data available compared to those previously reported [9]. Aided pure tone audiometry (at 0.5, 1, 2, and 4 kHz) and speech audiometry data, viz., speech recognition threshold (SRT) and word recognition score (WRS) at 65 dB HL, were collected with the update fittings. All audiometric tests were conducted in the sound field with the normal ear plugged and covered with an earmuff. Baseline audiometry was performed with headphones.

### Sound localization

Sound localization was tested with the minimum audible angle test (MAA; see Dun et al. [18]) and with a localization setup described by Agterberg et al. [19, 20]. The MAA test was used to examine the minimal angle at which two speakers could be discriminated. In this test, the patients were seated comfortably in a chair in a sound-attenuated booth, with two speakers at 90° azimuth left and right. A broadband noise burst of 0.3 s with roved amplitudes was randomly presented from one of both speakers. If the subject identified the speaker from which the stimulus was presented, the angle was changed to 60° azimuth. If sounds were again identified properly, the angle was further reduced (subsequently to 30°, 15°, 10°, and 5°). The smallest angle at which all sounds are identified correctly is the MAA. Tests were conducted late 2011 and early 2012. Five normal hearing children participated in the MAA as part of school project, which provided a reference.

The MAA test provides information about the ability to lateralize sound. To measure the localization abilities of the patients we applied another test setup. Broadband noise stimuli (0.5–20 kHz; *n* = 36) were presented in a completely dark, sound-attenuated room, to ensure that patients could only use acoustic information to localize sounds. Sound levels ranged between 45 and 65 dB SPL in broadband and duration of all stimuli was 150 ms. The subjects indicated the direction of the sound (ranging from −85° to +85° in azimuth) by means of head movement (magnetic search-coil induction technique) with a laser pointer mounted on eyeglasses pointed on a small plastic frame in front of them. The test setup [21, 22] and the processing of the data [19, 20] are described in detail in previous studies. Recently, this setup and the test protocol were also used for testing sound localization in children [20]. The outcome of this test is defined by the best linear fit of the stimulus–response relationship on the azimuth data, which is derived from the following formula:

$$a_{\text{RESP}} = b + g \cdot a_{\text{STIM}}$$

in which *a* is the azimuth angle (in degree), *b* is the response bias (in degree), and *g* the response gain (dimensionless). Furthermore, the mean absolute error (MAE) was calculated. Tests were conducted over the course of 2012 and 2013.

## Results

### Tolerability and appreciation

No complications occurred during surgery. The surgery time for the Sophono was slightly longer compared to the BAHA implantation, however, consisted of a one-stage procedure, compared to a two-stage procedure in most BAHA patients. The mean follow-up of the patients with the Sophono at the time of the retrospective data analysis was 3.6 years (range 3.1–4.7 years). Two of the six patients stopped using their device: patient 2 after approximately 1.5 years because of cosmetics and patient 6 after approximately 3 years because she experienced too little audiological benefit. All other patients reported to use their device predominantly at school. Few skin complications were reported. Some patients experienced minor discomfort due to pressure after wearing the device continuously, which was quickly resolved after removing the sound processor for a short time. At physical examination during regular follow-up visits to our clinic, once a crust on the skin at the implant site was detected, without the patient experiencing any symptoms. The surrounding skin was not infected. Therefore, this was treated conservatively with fusidic acid cream for a week. No implant loss, serious skin infections, or reasons for revision surgery were encountered. One patient underwent an abdominal MRI scan (1.5 tesla) without complications at the implant site. Overall the remaining four patients reported positively on the benefit they experienced when using the device.

The mean follow-up of the patients in the BAHA group was 4.7 years (range 3.2–5.9 years). Just like in the Sophono users, two children stopped using the device. Moreover, they had their implant or abutment removed, both after 4.5 years. They reported to experience too little hearing benefit. Another patient was lost to follow-up, as he moved to China and we were not able to check whether he was still using the device. Besides the previously reported re-implantation in one patient [9] and one patient that presented with postoperative hematoma, five patients experienced at least mild soft tissue reactions (Holgers grade 1) at some point during follow-up. Two patients presented with Holgers grade 2 soft tissue reactions at some time during follow-up that were easily and conservatively treated with antibiotic ointment. One patient reported soft tissue reactions to occur four times a year during follow-up over telephone. These reactions could not be specified to a Holgers grade, as she did not visit our clinic for evaluation and treatment of each soft tissue reaction, yet did not request treatment on the other hand.

### Audiometry

Table 1 presents audiometry details for all tested patients. At the time of these audiometrical evaluations, patient 2 already quit using the device. The aided audiometry with the first-generation sound processor (Alpha 1) was conducted at a mean follow-up time of 1.5 years (range 1.0–2.6 years) and the measurements with the second-generation sound processor (Alpha 2) were performed approximately 6 months later. The measurements with the first-generation sound processor differ slightly from earlier reported outcome [9], measured shortly after implantation, also due to updated fitting software. As illustrated by Table 1, audiometry results with either generation sound processor are comparable.

**Table 1:** Patient characteristics and audiometry data of the affected ear

Patient	Age	Gender	Atresia	BCD	Baseline				Aided (previously reported [9])			Aided (first generation, updated fitting software)			Aided (second generation)		
					PTA AC	PTA BC	SRT	WRS	PTA	SRT	WRS	PTA	SRT	WRS	PTA	SRT	WRS
1	5	M	IIB/III AD	Sophono	56	11	67	0	40	29	92	29	22	97	24	25	90
2	10	F	III AS	Sophono	61	0	60	0	30	27	92	n.a	n.a	n.a	n.a.	n.a.	n.a.
3	6	M	III AD	Sophono	63	4	n.a.	n.a.	35	29	90	25	15	100	23	25	93
4	5	M	III AS	Sophono	60	4	65	0	41	31	72	31	34	70	41	n.a.	n.a.
5	11	M	IIIAD	Sophono	54	9	n.a.	n.a.	36	29	90	35	27	90	34	27	92
6	7	F	OCA AS	Sophono	53	11	38	92	36	35	70	35	25	100	36	27	90
Mean	7				58	7	58	23	36	30	84	31	25	91	32	26	91
7	8	M	III AS	Divino	68	9	65	0	38	30	90						
8	9	M	III AS	Divino	66	13	63	0	39	30	92						
9	10	F	III AS	BP100	74	14	53	10	24	23	97						
10	6	M	III AS	Divino	78	20	70	0	35	12	96						
11	8	M	III AD	Divino	61	9	63	0	n.a.	25	78						
12	8	F	III AD	Divino	69	10	70	0	30	20	92						
Mean	8				69	12	64	2	33	23	91						

These data have partially been published earlier in Hol et al. [9]

*Age* Age at implant surgery; gender: *M* male; *F* female; *AD* right ear; *AS* left ear; *OCA* ossicular chain anomaly; *PTA* pure tone average in dB HL at 0.5, 1, 2, and 4 kHz; *AC* air conduction; *PTA BC* mean bone conduction thresholds in dB HL at 0.5, 1, 2, and 4 kHz; *SRT* speech recognition threshold in dB HL; *WRS* word recognition score at 65 dB HL in percent; *n.a*. data not available

### Sound localization

The MAA-results in Table 2 show better aided than unaided scores for both the Sophono users and the BAHA users. Three of the Sophono users already displayed good unaided MAA scores, which did not improve in the aided condition. All BAHA users showed improvement from their poor unaided scores, although they did not all meet the scores of the Sophono users. All normal hearing children reached the smallest possible MAA of 5°.

**Table 2:** The outcomes of the minimum audible angle (MAA) test

Patient	Unaided	Aided
1	15	15
2	15	15
3	90	20
4	90	15
5	90	10
6	10	10
Mean	52	14
7	90	15
8	90	30
9	60	10
10	60	20
11	90	30
12	90	30
Mean	80	23

Normal hearing children all scored a MAA of 5°. Patient numbers correspond to the data in Table 1

**Fig. 1:**
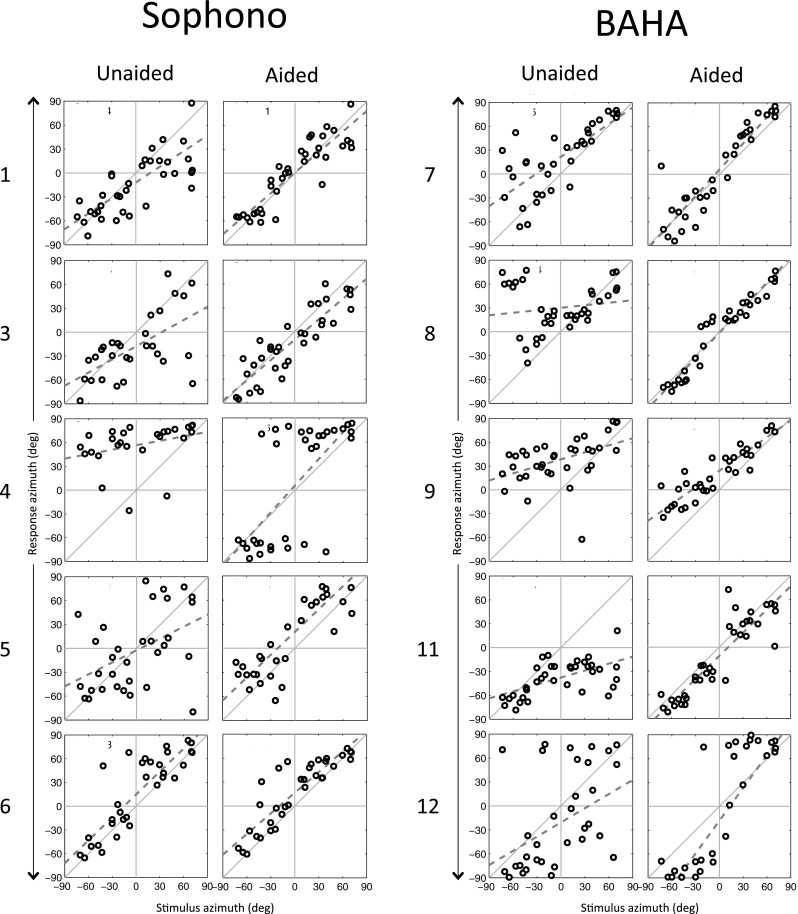
The outcomes of the sound localization test. Localization abilities in the unaided condition are compared to the aided condition using either the Sophono or the BAHA. Patient numbers correspond to the data in Table 1

Figure 1 shows the stimulus response relationships of five of the Sophono users (left column) and five of the BAHA users (right column). The individual plots demonstrate that all BCD users’ sound localization abilities improved in the aided condition as compared to the unaided condition; the aided data plots approach the diagonal more closely than the unaided data plots. However, patient 6 showed only a small improvement because the unaided localization ability was already reasonable good. Despite the clear improvement of the localization abilities of patient 4, this patient demonstrated lateralization instead of localization of the stimuli.

**Fig. 2:**
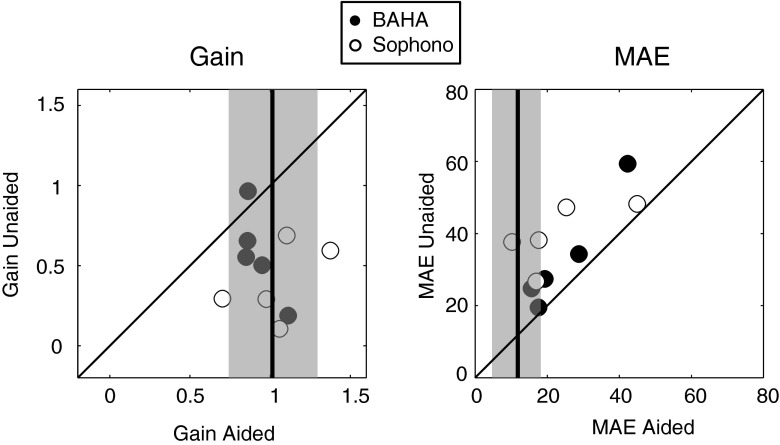
Comparison of the gain and the MAE of the sound localization test in the aided condition to the unaided condition. Concerning gain: 0 corresponds to no sound localization, whereas 1 corresponds to perfect localization. Concerning MAE: this represents the mean absolute difference between the target position and the actual response

In Fig. 2, for each of the patients the aided gain and MAE are plotted against the unaided values. This figure demonstrates the improvement in the aided condition compared to the unaided condition, since the data points are below the diagonal in the gain plot (gain in the aided condition is closer to 1 than the gain in the unaided condition) and the data points in the MAE plot are above the diagonal (i.e., smaller, thus, better MAE in the aided condition than in the unaided condition).

## Discussion

In the current study, we presented data obtained with at least three years of follow-up on a population with congenital conductive UHL who were rehabilitated with either a Sophono or a BAHA. The first audiological comparison between these BCDs in the same patients was reported previously [9]. Four out of six patients implanted with the Sophono were still using their device after a mean follow-up time of 3.6 years, mainly at school. The same applies to the BAHA users at a mean follow-up of 4.7 years, although one patient’s use could not be confirmed. Soft tissue tolerability with the Sophono was favorable. Subjective appreciation of the users was comparably reasonable. The updated second-generation transcutaneous sound processor (Alpha 2) provided similar hearing outcome compared to the first-generation sound processor (Alpha 1). To evaluate binaural restoration, sound localization was tested. Both groups of BCD users showed improvement in sound localization compared to the unaided situation.

The main strengths of the current study are the homogeneous populations and the relatively long follow-up compared to other studies, which is discussed in the next section. Furthermore, unique data concerning sound localization are presented. Sound localization is considered a major outcome measure of this intervention. The limitations of the current study are the small study populations with high dropout rates. Besides, the retrospective study design is methodologically weaker than a prospective (randomized controlled) clinical trial would have been.

The first case series that have been published on the Sophono studied populations with diverse types of hearing loss. Some case series present data of populations with both conductive and mixed hearing loss [23, 24] while others provide only concise audiometry data [25, 26]. Case series that consist of patients with conductive hearing loss, included both unilaterally and bilaterally affected patients [10, 27–29]. Because of these mixed populations, it is hard to substantially objectify or compare the audiological outcomes. Soft tissue outcomes have been generally reported positive.

Due to the regular follow-up visits and standardized procedures with extensive audiometry and sound localization testing, the results are considered to provide substantial information on both types of BCDs. In contrast to the literature that has already been published on the Sophono, the current results are derived from a homogeneous population with a comparison population using a BAHA over a relatively long-term follow-up period.

The clinical outcome of the current case series with the Sophono is considered favorable to that of the BAHA when applied in children. It has been demonstrated that 15.2 % of the previous generation percutaneous implants are lost in children and adverse skin reaction were found in 7.8 % of follow-up visits, which is considerably lower in adults [11]. Arguably, in adult patients, skin reactions with percutaneous implants are decreasing to such low values with updated implant designs [12, 13], that no significant difference between soft tissue tolerability between a transcutaneous and percutaneous BCD is to be expected. Furthermore, in children, implant survival is reported to increase with these new implants: implant loss of 3.5 % is reported in children with the wider diameter implants [14]. In contrary, soft tissue problems with the Sophono were encountered in another study, with 5 of 14 implants (36 %) having significant enough difficulties to discontinue use for a certain period [26].

Only four of the original six implanted patients in either group were still using their BCD. It has recently been found that long-term compliance with a BCD (although percutaneously applied in that study) in congenital conductive UHL patients was disappointing [30]. However, follow-up time in the current study is shorter and the study population is considerably smaller. Nevertheless, it could therefore be speculated that this specific indication is a challenging handicap to maintain satisfaction and device use over time.

Aided threshold in pure tone audiometry were around 30 dB HL in the affected ears, despite extensive fitting and testing in case of the Sophono group. These outcomes are comparable to the results of another study with a comparable population [29]. We did not perform additional audiometry in the BAHA group compared to the previous report on this population. However, these aided thresholds were also not as good as we had expected, especially concerning the outcomes with the skull simulator [9]. Nevertheless, the BAHA group showed larger improvement in hearing compared to the Sophono group. We consider these thresholds unsatisfactory; in patients with normal cochlear function aided thresholds with a properly functioning BCD should ideally be around 10–20 dB HL [6, 31]. As a result, the use of head shadow, binaural squelch, and binaural summation might be below expected values.

Sound localization is an important feature of binaural hearing [32]. Treatment of conductive UHL ideally provides access to binaural cues. Generally, the present outcomes demonstrated good aided localization abilities in both tests. However, in adults with acquired conductive UHL the application of a BAHA improves sound localization more clearly [19, 33]. On the other hand, remarkably good monaural localization abilities were found in the test population. It has been reported previously that some patients with congenital conductive UHL perform well on different sound localization tests [33, 34]. Possibly, patients with congenital conductive UHL have developed a different strategy for directional hearing, as they have coped with unilateral hearing all their life. Furthermore, we cannot exclude that longer periods of BCD use affect the directional hearing abilities. Good MAA scores with unilateral input have also been reported for children with bilateral conductive hearing loss by Dun et al. [18]. Despite fair to good improvement found in the MAA and sound localization test in the current population, localization abilities of children treated with a BCD for congenital conductive UHL do not reach the localization abilities of children with normal hearing [20].

## Conclusion

After at least 3 years of follow-up, soft tissue tolerability was favorable in children implanted with a Sophono compared to a BAHA. Aided thresholds with the Sophono were judged unsatisfactory, also with the updated second-generation sound processor. Sound localization improved with either BCD in these children with congenital conductive UHL, although the aided localization performance was not as good as in normal hearing children. Although not specifically examined in the current study, also differences in surgery and MRI issues need to be taken into account. The MRI compatibility of and image scattering caused by the Sophono as well as its lower output (as measured on a skull simulator) compared to the BAHA are important when counseling the patient and its caretakers. Based on the previous, the selection of a suitable amplification option should always be made deliberately and on individual basis for each patient, especially in this diverse group of children with congenital conductive UHL.
